# Beyond Domestication: Occurrence of Levator Anguli Oculi Medialis and Retractor Anguli Oculi Lateralis in Four Neotropical Canid Species

**DOI:** 10.1111/ahe.70121

**Published:** 2026-05-02

**Authors:** Paulo de Souza‐Junior, Erick Candiota Souza, Wilson Viotto‐Souza, Maria Eduarda Rodrigues Costa, Amanda Zanesco Crivelaro, Carlos Benhur Kasper, Marcelo Abidu‐Figueiredo

**Affiliations:** ^1^ Laboratory of Animal Anatomy Universidade Federal Do Pampa (UNIPAMPA), Programa de Pós‐Graduação Em Ciência Animal (PPGCA) Uruguaiana Rio Grande do Sul Brazil; ^2^ Fundação Presidente Antônio Carlos (UNIPAC) Uberlândia Minas Gerais Brazil; ^3^ Laboratory of Biology of Mammals and Birds Universidade Federal Do Pampa (UNIPAMPA), Programa de Pós‐Graduação Em Ciências Biológicas (PPGCB) São Gabriel Rio Grande do Sul Brazil; ^4^ Department of Animal and Human Anatomy Universidade Federal Rural Do Rio de Janeiro (UFRRJ) Seropédica Rio de Janeiro Brazil

**Keywords:** Carnivora, comparative anatomy, evolutionary adaptation, facial expressions, orbital musculature

## Abstract

The facial mimetic muscles levator anguli oculi medialis (LAOM) and retractor anguli oculi lateralis (RAOL) have been associated with expressive eye movements in canids and proposed as products of domestication. We investigated their occurrence in four Neotropical species ( *Lycalopex gymnocercus* , *Lycalopex vetulus* , *Cerdocyon thous* and *Chrysocyon brachyurus* ). Twelve adult specimens were dissected, and both muscles were consistently identified in all taxa, with conserved architecture. These findings support the interpretation that LAOM and RAOL are not exclusive to domestic dogs and may represent a broadly conserved trait across Canidae.

## 1 Introduction

The mimetic muscles of the orbital region, particularly the levator anguli oculi medialis (LAOM) and the retractor anguli oculi lateralis (RAOL), have received increasing attention due to their association with expressive facial movements in canids. In domestic dogs, the LAOM is described as a thin, flattened muscle band located in the dorsomedial orbital region, originating from the aponeurosis of the frontalis and frontoscutularis muscles and inserting into the fascia and skin at the medial canthus of the eye, in continuity with the orbicularis oculi (Hermanson 2020). Its contraction elevates the medial angle of the upper eyelid and palpebral hairs (Budras et al. 2007), constituting the anatomical basis of the expression AU101 in DogFACS (Waller et al. 2013). AU101 corresponds to the inner brow raising, in which elevation of the medial upper eyelid and adjacent brow region produces a more rounded ocular appearance (Waller et al. 2013). The RAOL, in turn, originates from the caudolateral orbital fascia near the zygomatic fossa and inserts into the lateral canthus of the palpebral fissure, retracting the eyelids caudolaterally and widening the palpebral fissure (Budras et al. 2007; Hermanson 2020).

A landmark study proposed that domestication favoured the development of these muscles in dogs, suggesting that the LAOM is well‐developed only in *Canis familiaris* , whereas it is vestigial or absent in *Canis lupus* , and that the RAOL is also less robust in wolves (Kaminski et al. 2019). This interpretation gained prominence by linking the emergence of these structures directly to human–dog communication (Mota‐Rojas et al. 2021). However, subsequent investigations challenged this view. Both LAOM and RAOL have been documented in wild canids, including coyotes ( *Canis latrans* ) and foxes ( *Urocyon cinereoargenteus* , *Vulpes vulpes* , *Vulpes lagopus* ), although their robustness and distinctiveness from the orbicularis oculi vary (Cunningham et al. 2024; Sexton et al. 2024). In the African wild dog ( *Lycaon pictus* ), both muscles are well developed and integrated into the orbitofrontal complex, further refuting their exclusivity to domestic dogs (Smith et al. 2024).

Moreover, histochemical analyses in mimetic muscles revealed a higher proportion of fast‐twitch fibres in dogs compared to slow‐twitch fibres in wolves, indicating that domestication may have shaped their physiology and performance rather than their anatomical emergence (Burrows et al. 2025). In this regard, Cunningham et al. (2024) demonstrated that *C. latrans* exhibits a well‐developed LAOM, in contrast to the reduced or absent condition in *C. lupus* , underscoring the importance of broader comparative approaches.

Despite the growing research on mimetic musculature in domestic and wild canids, Neotropical species remain largely overlooked in anatomical studies of this kind. This study investigates the occurrence and morphology of these muscles in four South American species ( *Chrysocyon brachyurus* , *Lycalopex gymnocercus* , *Lycalopex vetulus* and *Cerdocyon thous* ) to contribute to a broader understanding of the evolutionary history and functional diversity of mimetic musculature within Canidae.

## 2 Materials and Methods

The sample consisted of 12 adult cadaveric specimens, including four *Lycalopex gymnocercus* (3 males and 1 female), two *Lycalopex vetulus* (1 male and 1 female), four *Cerdocyon thous* (2 males and 2 females) and two *Chrysocyon brachyurus* (2 males), collected as roadkill in the states of Rio Grande do Sul and Minas Gerais, Brazil (authorisation IBAMA SISBIO 33667 and cooperation agreement IBAMA/UFU 002/2011, respectively). Only individuals with preserved soft tissues in their heads were included in the study. All carcasses had been fixed in 10% formaldehyde solution.

The skin of the facial region was incised and reflected, preserving the superficial fascia and the underlying mimetic musculature. Dissection focused on the orbital region, with gradual removal of the subcutaneous tissue under magnification. The LAOM was examined in the dorsomedial portion of the orbit, and the RAOL in the caudolateral orbital region. Careful dissection was required to distinguish both muscles from adjacent fibres of the orbicularis oculi.

## 3 Results

**FIGURE 1:**
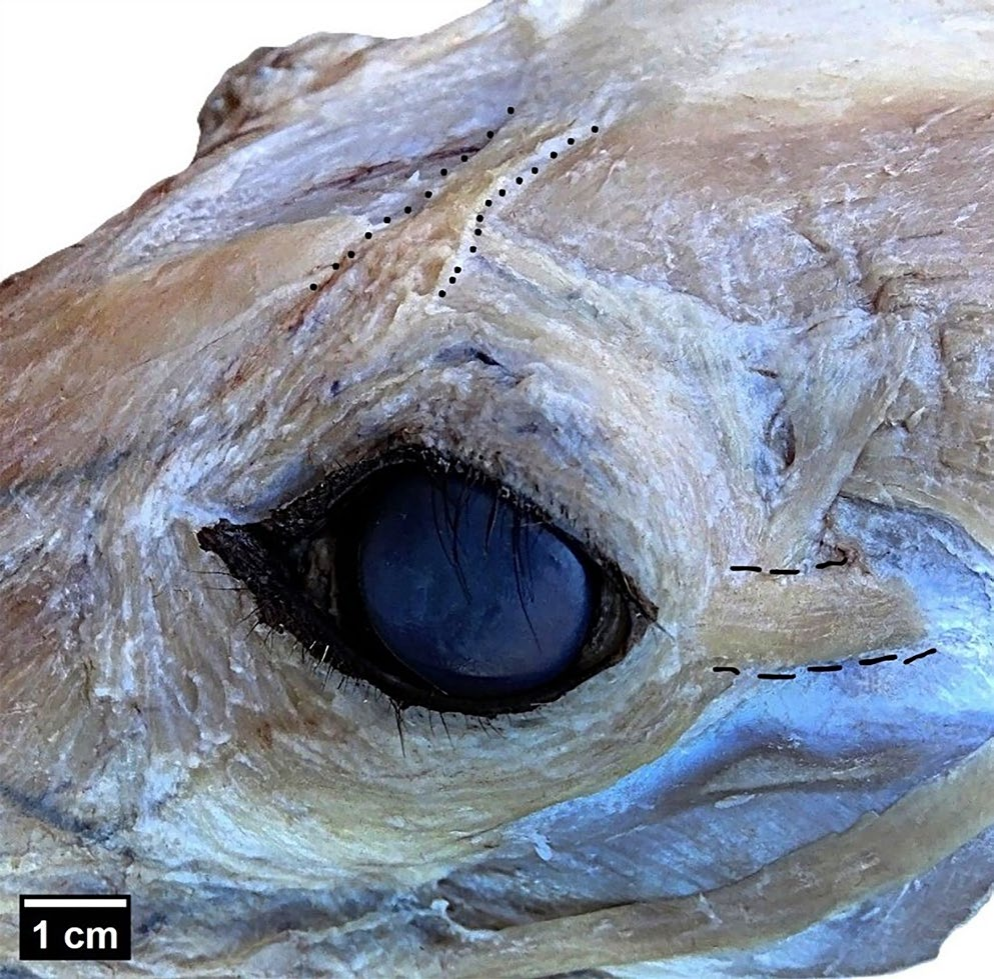
Photomacrograph in dorsolateral view of the left antimere showing the levator anguli oculi medialis (LAOM, dotted line) and the retractor anguli oculi lateralis (RAOL, dashed line) in an adult *Lycalopex gymnocercus* specimen. Scale bar: 1 cm.

**FIGURE 2:**
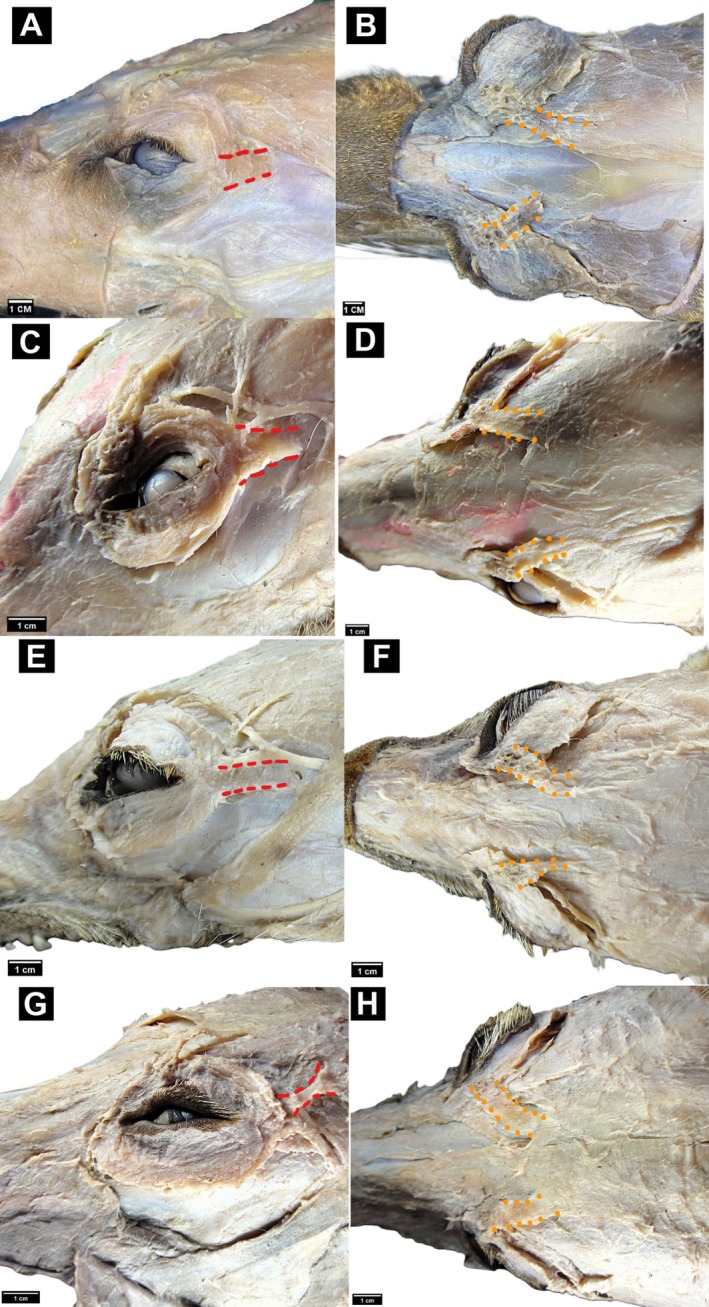
Photomacrographs in lateral view (left) and dorsal view (right) of the facial musculature dissection in Neotropical canids. *Levator anguli oculi medialis* (LAOM, dotted red lines) and *retractor anguli oculi lateralis* (RAOL, dashed orange line) highlighted in *Lycalopex gymnocercus* (A) lateral and (B) dorsal views; *Cerdocyon thous* (C) lateral and (D) dorsal views; *Lycalopex vetulus* (E) lateral and (F) dorsal views; *Chrysocyon brachyurus* (G) lateral and (H) dorsal views. Scale bar: 1 cm.

The presence of the retractor anguli oculi lateralis (RAOL) and the levator anguli oculi medialis (LAOM) was confirmed in all 12 specimens examined (Figures 1 and 2). In all samples, the LAOM was identified in the dorsomedial orbital region, in close relation to the frontalis and frontoscutularis muscles, and extended toward the medial canthus, where it blended with the orbicularis oculi. The RAOL was identified in the caudolateral orbital region, between the zygomatic arch and the temporal fascia, and extended toward the lateral angle of the eyelids, where it interlaced with the orbicularis oculi and adjacent skin.

During dissection, the LAOM in *L. vetulus* appeared slightly less conspicuous in gross view than in the other canids examined. Likewise, the RAOL in *C. brachyurus* appeared less conspicuous during gross dissection. These observations were qualitative and were not based on standardised morphometric measurements.

## 4 Discussion

The consistent identification of the LAOM and the RAOL in four Neotropical canid species examined reinforces the interpretation that these structures are not products of domestication, but rather components broadly conserved within the ancestral muscular repertoire of the family Canidae (Cunningham et al. 2024; Sexton et al. 2024; Smith et al. 2024). By documenting their occurrence in South American canids, our findings expand the comparative anatomical framework for mimetic musculature in Carnivora and strengthen the view that these muscles likely predate dog domestication. Considering that the South American canid lineage diverged from the wolf–dog lineage approximately 9–10 million years ago (Lindblad‐Toh et al. 2005), the presence of both muscles in all studied taxa is more consistent with a widespread canid condition than with a recent anatomical novelty restricted to domestic dogs. In this context, the reduced or absent condition previously reported for grey wolves (Kaminski et al. 2019) may represent a derived modification within the family, although broader taxonomic sampling would be necessary to evaluate this hypothesis more rigorously (Cunningham et al. 2024; Sexton et al. 2024). In all four species, the LAOM and RAOL showed a topographic arrangement comparable to that described for domestic carnivores by Hermanson (2020), reinforcing the interpretation that these structures are broadly conserved within Canidae.

The subtle interspecific variation observed during gross dissection may add an ecological dimension to the interpretation. In the examined material, the LAOM in *L. vetulus* and the RAOL in *C. brachyurus* appeared slightly less conspicuous than in the other species. Because no standardised morphometric measurements were performed, these observations should be interpreted cautiously. Notably, the two species in which these qualitative differences were noted are associated with the Cerrado habitat, whereas the other taxa are more closely associated with Pampas grasslands. These ecological differences may provide relevant context for future comparative studies, particularly because the species studied also differ in dietary profiles and habitat use (Table 1) (Berta 1982; Bossi et al. 2019; Dietz 1984; Kasper et al. 2016; Lucherini 2016). Although these ecological considerations do not exclude the influence of phylogenetic history, they suggest that, despite the conserved basic architecture of the LAOM and RAOL, their gross morphology may also be shaped by ecological and behavioural pressures. These scenarios remain speculative and should be interpreted cautiously.

**TABLE 1:** Comparative summary of phylogenetic context, sample size, ecology and gross qualitative expression of the LAOM and RAOL in the four Neotropical canids examined.

Scientific name	Phylogenetic context^a^	*n*	Ecological/dietary category	LAOM^b^	RAOL^b^
*Lycalopex gymnocercus*	*Lycalopex* lineage (South American foxes)	4	Generalist	Conspicuous	Conspicuous
*Lycalopex vetulus*	*Lycalopex* lineage (South American foxes)	2	Predominantly insectivorous	Less conspicuous	Conspicuous
*Cerdocyon thous*	South American lineage, sister group of *Lycalopex* group	4	Generalist	Conspicuous	Conspicuous
*Chrysocyon brachyurus*	South American lineage, sister group of *Cerdocyon* and *Lycalopex* groups	2	Omnivorous, with high fruit intake	Conspicuous	Less conspicuous

^a^ Based on Chavez et al. (2022).

^b^ Qualitative categories based on gross anatomical observation during dissection; no standardised morphometric measurements were performed.

The LAOM should also be interpreted within a broader orbitofrontal context rather than as an isolated structure. In domestic dogs, this muscle arises from the aponeurotic contribution of the frontalis and frontoscutularis muscles (Hermanson 2020), and in wild canids its functional significance may likewise depend on integration with adjacent frontal and auricular musculature. This broader anatomical perspective is reinforced by comparative evidence from *L. pictus* , in which the mimetic and auricular muscles of the orbital region were interpreted as part of an integrated orbitofrontal complex associated with social signalling (Smith et al. 2024). In the present study, however, our dissections were directed primarily toward identifying the LAOM and RAOL and did not include a comparative assessment of the relative development of the frontalis, frontoscutularis, or associated auricular muscles. For this reason, any inference about coordinated variation within this complex should be considered tentative.

At the functional level, multiple lines of evidence suggest that the mere presence of these muscles does not guarantee direct communicative function. Histochemical studies have shown that dogs possess a predominance of fast‐twitch fibres in their facial muscles, favouring rapid expressions and pulsatile vocalisations such as barking, whereas wolves display a greater proportion of slow‐twitch fibres, which are suited to sustained contractions supporting prolonged howling (Burrows et al. 2025). This pattern suggests that the relative reduction of LAOM and RAOL in wolves may represent a physiological adaptation that favours acoustic signalling over rapid facial movements (Burrows et al. 2025; Kaminski et al. 2019; Waller et al. 2013). Additionally, the myosin distribution pattern of a Siberian Husky specimen revealed a low proportion of slow fibres, resembling the wolf profile (Burrows et al. 2025). This finding may be significant because the Siberian Husky is considered one of the most ancient dog breeds, genetically closer to wolves than most modern breeds (Bergström et al. 2020; Lehoczki et al. 2023), underscoring evolutionary continuity between wolves and early domestic lineages.

Morphophysiological evidence indicates that the mere presence of the LAOM and RAOL does not, by itself, guarantee a communicative function. From a behavioural perspective, Bremhorst et al. (2021) demonstrated that the AU101 expression, associated with the LAOM, occurs even more frequently in non‐social contexts and coincides with eye bulb movements in 94% of cases, suggesting that its activation may reflect biomechanical adjustments rather than intentional communication. Electromyographic recordings further support this view, showing that LAOM activity, along with that of other facial muscles, can be triggered by reflexes, visual attention and internal emotional states and that facial expressions result from the coordinated co‐activation of multiple muscles (Hobkirk and Twiss 2024). Capitain et al. (2025) reported that socialised dogs employed AU101 for longer than wolves during human interactions, but more striking differences were observed in auricular positions, with wolves maintaining erect ears and dogs showing rotated or lowered ears, often linked to submission or appeasement. Furthermore, the consistent human bias in expressing more positive signals toward dogs than wolves highlights that the function of these expressions must be interpreted within a bidirectional relational context.

It is also important to acknowledge that methodological differences across studies may influence the identification of mimetic muscles. Specimen preservation, fixation type (formaldehyde vs. freezing) and the dissector's experience handling delicate fibres can affect the distinction between LAOM and RAOL, contributing to divergent interpretations in the literature.

This study has some limitations that should be acknowledged. First, although the consistent identification of the LAOM and RAOL across all specimens supports their occurrence in the four species examined, the limited sample size, particularly for taxa represented by only two individuals, does not permit a comprehensive assessment of intraspecific variation. Second, although all specimens were classified as adults, exact age determination was not possible and potential effects of age‐related variation or sexual dimorphism could not be evaluated. Third, because the present study was based on gross anatomical dissection, the macroscopic presence of these muscles does not by itself demonstrate a primary communicative function. In particular, without histological or histochemical assessment of muscle fibre composition, any functional interpretation regarding rapid facial expression or communicative specialisation must remain provisional.

Taken together, our findings indicate that the LAOM and RAOL are present in four Neotropical canids and reinforce the interpretation that these muscles are not exclusive to domestic dogs. More broadly, these results expand the comparative anatomical framework of mimetic musculature in Canidae and highlight the need for integrative studies combining gross anatomy, histology and behaviour to clarify the functional significance of these structures.

## Funding

This study was financed in part by the Coordenação de Aperfeiçoamento de Pessoal de Nível Superior—Brasil (CAPES)—Finance Code 001.

## Conflicts of Interest

The authors declare no conflicts of interest.

## Data Availability

The data that support the findings of this study are available from the corresponding author upon reasonable request.
